# Early Detection of Locomotion Disorders in Gilts Using a Novel Visual Analogue Scale; Associations with Chronic Stress and Reproduction

**DOI:** 10.3390/ani11102900

**Published:** 2021-10-06

**Authors:** Martyna E. Lagoda, Laura A. Boyle, Joanna Marchewka, Keelin O’Driscoll

**Affiliations:** 1 Pig Development Department, Teagasc Animal and Grassland Research and Innovation Centre, Moorepark, Fermoy, Co., P61 P302 Cork, Ireland; laura.boyle@teagasc.ie (L.A.B.); keelin.odriscoll@teagasc.ie (K.O.); 2 Department of Animal Behaviour, Institute of Genetics and Animal Biotechnology of the Polish Academy of Sciences, ul. Postępu 36A, Jastrzębiec, 05-552 Warsaw, Poland; J.marchewka@igbzpan.pl

**Keywords:** swine, mobility, lameness, welfare, productivity, cortisol

## Abstract

**Simple Summary:**

Lameness in sows causes pain and poor welfare. Early detection is crucial if treatment is likely to be effective. Locomotion scoring is the best way to achieve this, but existing scoring systems are not sensitive enough to detect subtle deviations from optimal locomotion. Our objective was to develop a new visual analogue scale (VAS) to measure the locomotory ability of sows over time. Effectiveness in detecting slight deviations was tested in young female pigs by comparing the scale to an existing categorical scoring system. The VAS detected slight deviations from optimal locomotion over time more effectively than the categorical locomotion scoring system. It was also positively associated with hair cortisol concentrations (chronic stress) and measures of reproductive performance. If used by farmers, the VAS could potentially help in lameness prevention and thereby improve sow welfare and performance.

**Abstract:**

Locomotion scoring is crucial for the early detection of lameness, which reduces sow welfare and performance. Our objective was to test the effectiveness of a visual analogue scale (VAS) to measure locomotory ability (OVERALL) compared to a categorical scoring system (CAT) and to investigate associations with hair cortisol and reproductive performance. Locomotion was scored in gilts (n = 51) at service, on day 57 and day 108 of pregnancy, and at weaning, using a VAS (150 mm line: 0 mm (perfect)–150 mm (severely lame)), and a CAT (1 (perfect)–5 (severely lame)). Hair cortisol concentration was measured on day 108 of pregnancy. Reproductive performance data (parity 1–4) were acquired from farm records. VAS detected deviations from optimal locomotion more effectively than the CAT (F_3,145_ = 2.70; *p* ≤ 0.05 versus F_3,195_ = 2.45; *p* = 0.065). Higher OVERALL scores at service (REG = 0.003 ± 0.0012; F_1,48_ = 4.25; *p* ≤ 0.05) and on day 57 (REG = 0.003 ± 0.0013; F_1,48_ = 6.95; *p* ≤ 0.05) were associated with higher hair cortisol concentrations on day 108. Positive associations were detected between OVERALL at service and the number of piglets born dead (REG = 0.01 ± 0.006; F_1,36_ = 4.24; *p* ≤ 0.05), and total born (REG = 0.1 ± 0.03; F_1,120_ = 4.88; *p* ≤ 0.05). The VAS better facilitates early detection of lameness, which could help to prevent detrimental effects, possibly mediated by chronic stress, on reproductive performance.

## 1. Introduction

Lameness is a painful, multifactorial disorder, considered one of the main welfare issues for sows [1,2,3]. It also has economic implications for the farmer, as it remains one of the primary reasons for premature culling of sows [1,2,3]. Chronic lameness [1] contributes to elevated stress levels (swine [4]; dairy cows [5]), and consequently, impaired reproductive performance [6,7]. For instance, lame sows had lower numbers of piglets born alive in the study of Anil et al. [8]. In dairy cows, O’Connor et al. [9] showed that even slight deviations from optimal locomotion can have a negative impact on reproductive performance parameters, such as calving interval, as well as cow death on farms and the reasons for slaughter. Moreover, the early detection of slight deviations from optimal locomotion is important, potentially acting as an early warning sign of a developing lameness disorder [1,3]. Early detection would allow the application of preventative lameness treatment at a stage when it is likely to be more effective [2,10], consequently reducing the associated chronic stress and the risks to reproductive performance.

There are several published sow locomotion scoring systems [11], with that of Main et al. [12] being the most commonly used. However, most are not detailed/sensitive enough to detect slight deviations from optimal locomotion [1,3], as in general they consist of categories clustering several descriptors together [1,3]. In addition, scoring systems often measure locomotion on an ordinal scale, despite the fact that locomotion traits can change in a continuous manner [13,14,15]. This can also lead to missing important variation in locomotion [15]. Taken together, this results in a reduced level of detail that a system can retain [1,3], and as such, an animal with a slight deviation from optimal locomotion could be classified as sound, because not all descriptors within a category are met [1]. Indeed, the sensitivity of a scoring system and therefore its ability to detect slight deviations from optimal locomotion is affected by the number of categories it possesses [1,16], with fewer categories meaning less sensitivity.

Even though the rationale for developing scoring systems with fewer categories was to improve inter-observer reliability [1,3], there is evidence that the reliability of more detailed scoring systems may actually be superior [3]. Moreover, a scoring system which has a larger number of categories, or is continuous, could potentially capture a slight deviation from optimal locomotion, when a less detailed scoring system may not [3]. In addition to scoring overall locomotory ability in a detailed manner, a scoring system could also consider individual locomotion components separately [17,18]. Based on dairy cow literature, such an approach facilitates better insight into an animal’s locomotory ability by revealing how different components may contribute to the overall locomotion score, and also aids in interpreting the causes of deviations from an ‘ideal’ stride [19,20,21].

Previous research suggests that visual analogue scales (VAS) could overcome these problems [1,3,15]. VASs assist human patients in rating their own pain experiences [22], and have been modified for use in animal locomotion assessment [13]. Many authors agree that VASs are more sensitive than categorical scoring systems, as they measure traits on a continuous scale, rather than restricting scores to discrete units [3,13,23]. Indeed, there is extensive use of VAS in the dairy cow literature [15,19,24,25]. There are advantages of measuring locomotion on a continuous scale when compared to categorical scoring systems [15,19]. However, to our knowledge, the use of a VAS in pigs is limited to two studies [3,26]. Thus, the aim of this study was to develop a novel VAS to assess both overall locomotory ability and individual aspects of gilt locomotion. We hypothesised that this VAS would allow us to (1) detect slight deviations from optimal gilt locomotion over time more effectively than a categorical scoring system; (2) identify a single component of locomotion which can provide a quick insight into the gilt’s overall locomotory ability; (3) detect chronic stress levels associated with impaired locomotion and predict reproductive performance of sows.

## 2. Materials and Methods

### 2.1. Ethical Approval

The research farm on which this experiment was conducted complied with Statutory Instrument number 311 of 2010 European Communities (Welfare of Farmed Animals) Regulations 2000. Experimental work was authorized by the Teagasc Animal Ethics Committee (Approval No: TAEC219-2019).

### 2.2. Animals and Housing

This study took place on a 200-sow research unit at the Teagasc Pig Development Department in Moorepark, Fermoy, Co. Cork, Ireland, between May 2019 and March 2020. In total, 51 gilts in eight replicate groups were used. Gilts were purchased from a breeder and thus had to undergo a six-week quarantine before entering the research unit at approximately 210 days of age. Upon completion of the quarantine period, gilts entered the main pig unit and were housed in fully slatted pens (3.2 m × 2.6 m) in groups of four, fed from a long-trough, and were treated with Altresyn for oestrus synchronisation. Gilts were served twice in service stalls by artificial insemination, first at the onset of standing oestrus, and then within 24 h. Each replicate was served between three to nine weeks apart, depending on the availability of new gilts entering the breeding pool as replacements (see Table 1 for experimental schedule). Approximately five days after service gilts were moved back into their home pens in the same groups as before service, where they stayed until day 30 of pregnancy.

They were then mixed into a larger dynamic group with other pregnant gilts (see Table 1 for number of gilts present at the time of mixing) where they were fed by an electronic sow feeder (ESF; Schauer Feeding System; Prambachkirchen, Austria) set to a 23 h cycle, starting at 17:00 daily. The ESF recognised each gilt by a transponder tag programmed to her individual daily allowance of a standard gilt diet. Water was available ad libitum from a single-bite drinker inside the ESF, and from a drinker bowl in the pen. The group pen (68.11 m^2^) comprised of fully slatted concrete floors in the group area, with four insulated solid concrete bays for lying. Gilts had a wooden block suspended from a chain as enrichment. Approximately one week prior to farrowing (day 108), they were moved to the farrowing accommodation and housed in standard individual farrowing crates (pen dimensions: 2.5 m × 1.8 m), with cast-iron fully slatted floors within the farrowing crate, plastic fully slatted floors around the crate, and a solid plastic heated mat for piglets. Weaning took place approximately 28 days post-partum.

### 2.3. Locomotion Scoring

Locomotion was scored visually while gilts walked on solid concrete along the corridor outside of the home pen, taking at least six strides (distance of approximately 30 m). Locomotion was scored on three occasions during the first pregnancy: three days before service (service), in mid-pregnancy (approximately day 57), and on the day of entry to the farrowing crates (day 108; late pregnancy). Sows were also scored at weaning of their first litter. Scoring was performed by a single trained observer who practiced until at least 90% intra-observer scores for repeatability were achieved.

#### 2.3.1. Categorical Locomotion Scoring (CAT)

Each gilt was assigned a locomotion score (0 to 5) using the gait component of the categorical locomotion scoring system developed by Main et al. [12].

#### 2.3.2. Visual Analogue Scales

- Overall locomotion scoring

**Figure 1:**
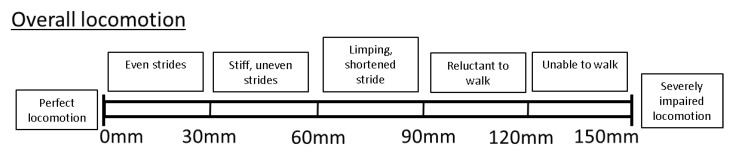
Example of a visual analogue scale for the scoring of overall locomotory ability developed for the purpose of this study.

Overall locomotory ability (OVERALL) was assessed using a VAS consisting of a 150 mm horizontal line, with the left end (0 mm) representing perfect locomotion, and the very right end (150 mm) representing severely impaired locomotion. Locomotory ability was scored by marking a point along the scale, with increasing impairment represented by a mark further to the right of the line. The distance from the left-hand end of the scale was measured and the value for each recorded in millimetres. Thus, the greater the number, the more impaired the locomotory ability. As a guide, the VAS was also divided into descriptive sublevels, to aid with consistency of locomotion scoring ([3,27,28]; e.g., Figure 1). The sublevels were selected based on previous literature on pig and dairy cow locomotion scoring [3,12,19,26].

- Component locomotion scoring

**Figure A1:**
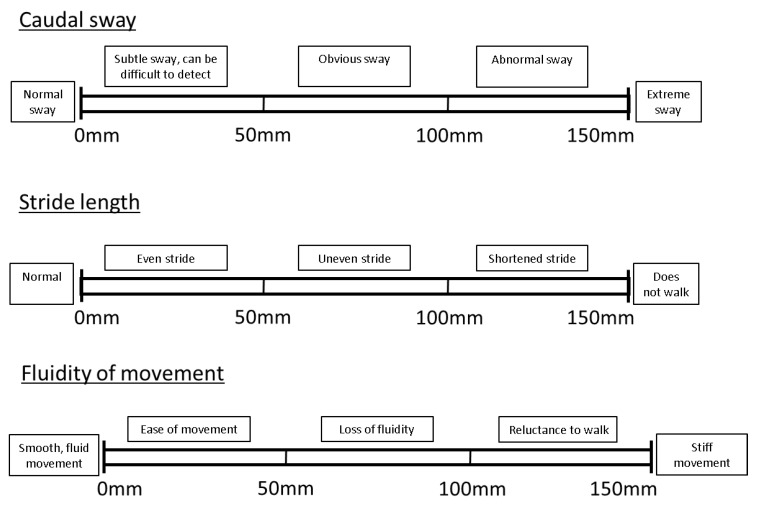

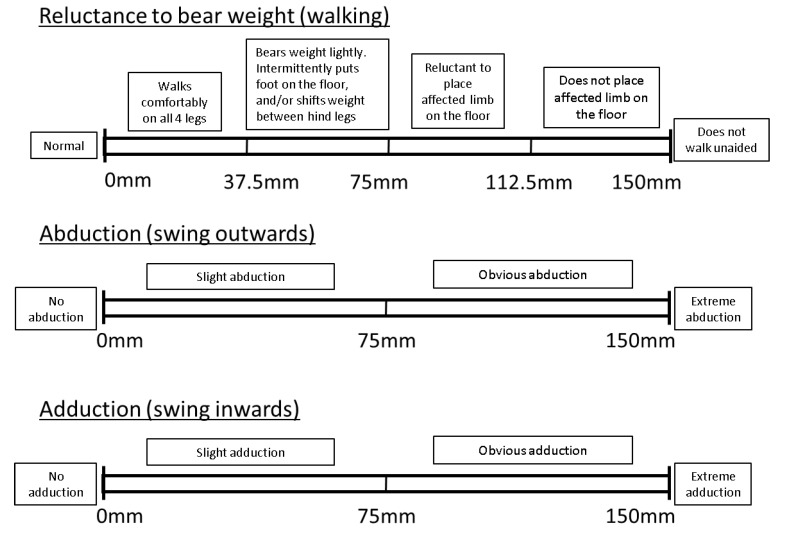
Individual visual analogue scale locomotion components, with descriptive sublevels based on pig locomotion assessment literature, to aid with consistency of scoring.

As well as the overall locomotory ability, several components of locomotion (Table 2) were assessed using an individual VAS for each component. These components were selected based on previous literature on pig and dairy cow locomotion scoring [3,12,19,26] and upon feedback gathered during a pilot trial whereby two authors (L.A.B. and K.O.) assessed locomotion in a number of sows. As in the case of OVERALL, the VAS for each of the individual locomotion components was also divided into descriptive sublevels to aid with consistency of scoring [3,12,19,26]. A different number of sublevels were applied to each locomotion component, based on severity levels reported on in the pig locomotion assessment literature ([3,26]; see Appendix A, Figure A1).

### 2.4. Hair Collection and Subsequent Hair Cortisol Concentration Analysis

Hair collection for cortisol determination was performed while gilts were inside the weighing scales immediately prior to mixing into the dynamic group (day 30 of pregnancy) and on the day of entry to the farrowing crates (day 108; late pregnancy) during their first pregnancy. Hair is hypothesised to be a suitable medium for quantifying chronic stress levels, due to the long-term accumulation of cortisol within the shaft [29,30,31]. Combined with this, the shave/re-shave method (first shave on day 30, then re-shave performed in late pregnancy) used in this study allowed determination of the concentration of cortisol which accumulated during the period between hair shavings. Thus, hair cortisol concentration measured in late pregnancy was used in the analysis as an indicator of chronic stress corresponding to approximately the last two-thirds of the pregnancy. The collection site can have an impact on cortisol concentrations found in hair [30,32], and thus, based on previous research, the dorso-lumbar region was selected as the most appropriate site for collection to best guarantee adequate measurement of cortisol concentration [32,33,34]. The dorso-lumbar site was identified by measuring 6.5 cm left and right from the mid-point at the spine marked by the position of the last rib; hair was shaved using an electric shaver, placed into plastic zip-lock bags, and frozen at −20 °C until hair cortisol analysis.

Hair sample preparation and cortisol extraction were based on the procedure described by Davenport et al. [29], with certain modifications described by Lagoda et al. [32]. In brief, hair samples were defrosted for one hour prior to preparation procedures, then washed by placing 300 mg of hair into a 10 mL polypropylene tube along with 5 mL of isopropanol, and mixing gently on a shaker for 3 min. This was repeated using fresh isopropanol for the second wash. Washed hair samples were left inside the wash tubes and placed inside a protected fume hood to dry overnight. Samples prepared in this way were then individually ground into a fine powder using a Retsch mixing mill (MM200; 10 mL stainless steel grinding jars, single 12 mm stainless steel grinding ball) for 4 min at 25 Hz. Approximately 50 mg of ground hair sample was weighed out and placed in a 2 mL tube along with 1 mL of methanol, which was followed by incubation of the sample for 24 h at room temperature with constant gentle agitation (approximately 95 rpm) for cortisol extraction. Following the 24 h incubation period, 0.6 mL of the cortisol extract in methanol was removed (taking care not to disturb the settled hair powder at the bottom of the tube) using an Eppendorf pipette and transferred to a clean 1.5 mL tube for methanol evaporation, which was performed using a stream of nitrogen gas at 38 °C. Cortisol extract samples were frozen at −20 °C pending EIA analysis. Extracted cortisol samples were analysed using Salimetrics^®^ Expanded Range, High Sensitivity Salivary Cortisol EIA kit, which was validated for the analysis of hair cortisol concentrations [29,33,35], and is valid for use in a range of species, including swine [29,34,36]. Frozen cortisol extract samples along with the EIA kit were brought to room temperature 1.5 h prior to being reconstituted with 0.4 mL of phosphate buffer (assay diluent) provided with the EIA kit. Reconstituted extracts (n = 102) were analysed for cortisol concentration levels in duplicate using 4 assays, following the protocol provided with the EIA kit. Inter- and intra-assay CV were 24.1 and 8.7%, respectively.

### 2.5. Reproductive Performance

Reproductive performance records were acquired from the sow management system (PigChamp) used on the farm, to ascertain the number of piglets born alive, born dead, mummified, and total born over four parities (parity 1 to 4).

### 2.6. Statistical Analysis

SAS v9.4 was used for all statistical analyses (SAS Inst. Inc., Cary, NC, USA) with sows as the experimental unit. Differences were reported when *p* ≤ 0.05, while statistical trends were reported when *p* > 0.05 and *p* ≤ 0.10. Results for independent continuous variables are reported as their regression coefficient (REG) ± standard error (SE).

#### 2.6.1. Comparison of Scoring Methods over Time

A repeated measures analysis was carried out to investigate the effect of time of locomotion scoring (n = 4) on locomotion scores recorded using OVERALL, locomotion components, and CAT. Residuals were checked for normality using the Shapiro test, and by examining the quantile-quantile plot. For variables with normally distributed residuals (OVERALL, and the components: caudal sway, stride length, fluidity of movement, and reluctance to bear weight while walking), linear mixed model equations were built in PROC MIXED. For variables with non-normally distributed residuals (CAT, and the components: abduction and adduction) generalised linear mixed model equations were built in PROC GLIMMIX and fitted with either the Poisson (abduction and adduction score) or the multinomial distribution (CAT). For model equations built in PROC MIXED, time was included as a repeated measure, with sow ID as subject, while for model equations built in PROC GLIMMIX, time was included as an additional random effect to account for repeated sow ID measures. Replicate was included as a random effect in all models.

#### 2.6.2. Associations between OVERALL and Locomotion Components

A repeated measures regression analysis was performed to investigate the association between OVERALL (dependent variable) and the individual components of locomotion (included as continuous independent variables; PROC MIXED) across all scoring days together, and also on each scoring day separately. The latter was completed as it is important to consider the relationship on the different days, since the changing shape and weight of the gilt with progressing pregnancy could potentially impact the way she walks. Residuals were checked as described previously to confirm the suitability of the models. Time was included as a repeated measure, and replicate was included as a random effect.

#### 2.6.3. Associations between OVERALL, Hair Cortisol Concentration, and Reproductive Performance

Separate regression analyses were performed to investigate the association between OVERALL at each of the three recording occasions during pregnancy, and hair cortisol concentration in late pregnancy. A separate regression analysis was also carried out to investigate the association between OVERALL at each of the three recording occasions and the following measures: the number of piglets born alive, born dead, mummified, and the total number born over four parities. Residuals were checked as before. Hair cortisol concentration, number of piglets born, and piglets born alive were analysed using linear mixed models (PROC MIXED), and the number of piglets mummified or born dead were analysed using generalised linear mixed models (PROC GLIMMIX) and fitted with the Poisson distribution. For the analysis of hair cortisol concentration, an EIA assay plate was included as an additional random effect. For the measures of reproductive performance which had model equations built in PROC MIXED, parity was included as a repeated measure, with sow ID as subject, while for model equations built in PROC GLIMMIX, parity was included as an additional random effect to account for repeated sow ID measures. Replicate was included as a random effect in all models.

## 3. Results

Gilts were considered lame if they received a score of 2 or higher (≥ 2) on the CAT scale (n = 5 gilts throughout entire study), and if they scored 60 mm or higher (≥ 60) on the VAS for OVERALL (based on the descriptive sublevel overlying the VAS, whereby visible signs of obvious lameness such as limping and shortened stride are described for the first time; n = 6 gilts throughout entire study). The mean ± standard deviation (SD) for the CAT locomotion score throughout the entire study was 0.2 ± 0.50 (median = 0; range 0 to 3). The mean ± SD for the OVERALL locomotion score throughout the entire study was 17.1 ± 14.47 mm (median = 15; range 1 to 72 mm).

### 3.1. Comparison of Scoring Methods over Time

**Figure 2:**
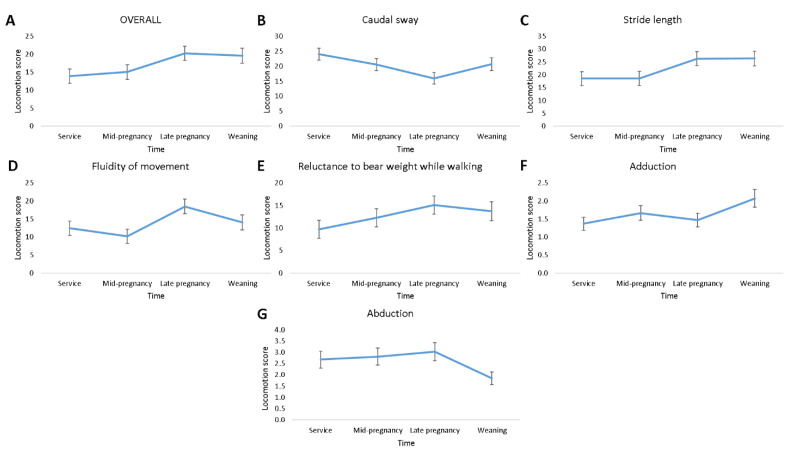
Plots of locomotion score least square mean ± standard error changes over time for OVERALL (**A**), caudal sway (**B**), stride length (**C**), fluidity of movement (**D**), reluctance to bear weight while walking (**E**), adduction (**F**), and abduction (**G**) in 51 gilts (n = 8 replicates).

**Figure 3:**
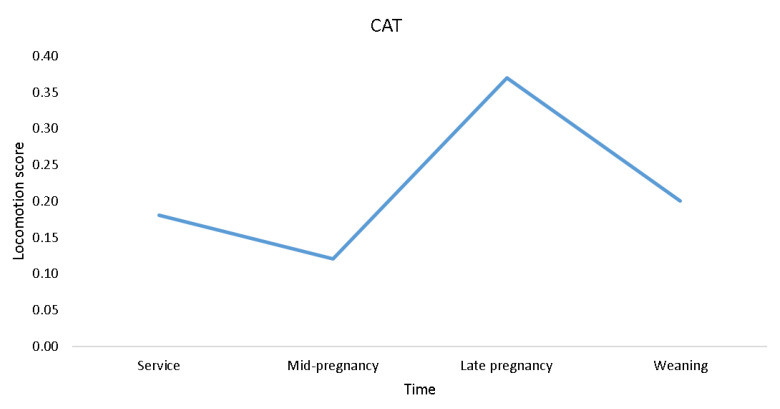
Plot of CAT mean locomotion score changes over time in 51 gilts (n = 8 replicates).

There was an effect of time of scoring on OVERALL (F_3,145_ = 2.70; *p* ≤ 0.05), and on some of the components of locomotion, namely, caudal sway (F_3,144_ = 2.92; *p* ≤ 0.05), stride length (F_3,145_ = 3.04; *p* ≤ 0.05), and fluidity of movement (F_3,145_ = 3.82; *p* ≤ 0.05; Figure 2). No effect of time of scoring on reluctance to bear weight while walking (*p* > 0.05) was found, while abduction (F_3,194_ = 2.47; *p* = 0.063) and adduction (F_3,194_ = 2.24; *p* = 0.086; Figure 2) tended to change over time. As shown in Figure 2, the pattern of locomotory ability over time which was most similar to OVERALL was that of stride length, with the least similar being caudal sway and abduction. Locomotion scores estimated using CAT tended to change over time (mean (median); at service = 0.18 (0); mid-pregnancy = 0.12 (0); late pregnancy = 0.37 (0); weaning = 0.20 (0); F_3,195_ = 2.45; *p* = 0.065; Figure 3).

### 3.2. Associations between OVERALL and Locomotion Components

**Figure A2:**
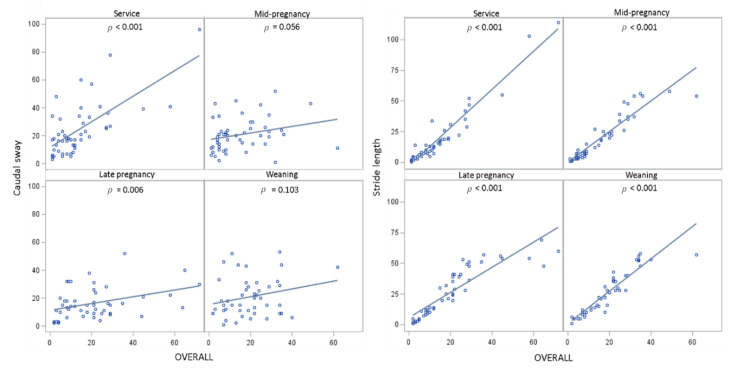

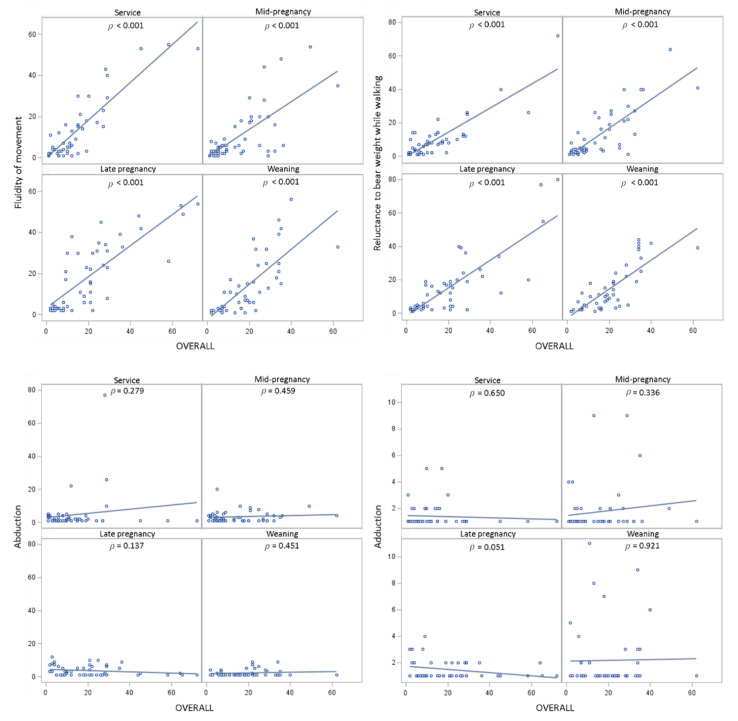
Graphs representing the relationship between OVERALL and locomotion components on each scoring day.

There were positive associations between the OVERALL VAS score and the scores for caudal sway, stride length, fluidity of movement, and reluctance to bear weight while walking across all scoring days together (Table 3), with the highest regression coefficients for the latter three measures. Indeed, although the association between caudal sway and OVERALL locomotion score across all scoring days together was positive, when considered on each scoring day separately, this association did not always hold true (e.g., service: *p* < 0.001; mid-pregnancy: *p* = 0.056; late pregnancy: *p* = 0.006; weaning: *p* = 0.103; see Appendix A, Figure A2 for graphs representing the relationship between OVERALL and locomotion components on each scoring day). This suggests that gilts with a higher OVERALL locomotion score also had higher caudal sway scores across all scoring days together, despite the pattern of increase/decrease being different for OVERALL and caudal sway locomotion scores on any given day. On the other hand, the associations between OVERALL locomotion score and stride length, fluidity of movement, and reluctance to bear weight while walking across all scoring days together were reflected by the associations found when each scoring day was considered separately (*p* < 0.001 for stride length, fluidity of movement, and reluctance to bear weight while walking on each scoring day; Appendix A, Figure A2).

### 3.3. Associations between OVERALL, Hair Cortisol Concentration, and Reproductive Performance

The OVERALL locomotion score both at service (REG = 0.003 ± 0.0012; F_1,48_ = 4.25; *p* ≤ 0.05) and at mid-pregnancy (REG = 0.003 ± 0.0013; F_1,48_ = 6.95; *p* ≤ 0.05) was positively associated with hair cortisol concentration in late pregnancy (i.e., the more impaired locomotory ability was during early to mid-pregnancy, the greater the accumulation of cortisol in the hair shaft by end of the pregnancy). No association between OVERALL locomotion score in late pregnancy and hair cortisol concentration in late pregnancy was found (*p* > 0.05).

The OVERALL locomotion score at service was positively associated with the number of piglets born dead (REG = 0.01 ± 0.006; F_1,36_ = 4.24; *p* ≤ 0.05), and the total born (REG = 0.1 ± 0.03; F_1,120_ = 4.88; *p* ≤ 0.05), and tended to be positively associated with the number of piglets born alive (REG = 0.1 ± 0.03; F_1,120_ = 3.17; *p* = 0.078) and piglets mummified (REG = 0.01 ± 0.008; F_1,24_ = 2.97; *p* = 0.098).

The OVERALL locomotion score in late pregnancy tended to be positively associated with the number of piglets born alive (REG = 0.04 ± 0.024; F_1,119_ = 3.06; *p* = 0.083) and total born (REG = 0.1 ± 0.03; F_1,119_ = 3.84; *p* = 0.053). There were no associations between OVERALL locomotion score at mid-pregnancy and any aspect of reproductive performance (*p* > 0.05).

## 4. Discussion

The detrimental nature of lameness [37] warrants the need for its early detection [2,10]. The VAS developed for the purpose of this study enabled the detection of slight deviations from optimal locomotion and its individual components over time, and as hypothesised, it was more effective at this than the categorical system developed by Main et al. [12]. Thus, it holds promise to be a more effective research tool than the categorical scale.

As expected, gilt locomotion scores increased as pregnancy progressed. This is because as pregnancy advances, gilts gain weight, which in turn puts more pressure on their limbs and could result in a deterioration in leg health and therefore higher locomotion scores [38]. Furthermore, the longer sows spend in a group, the greater the likelihood of fights and consequent injuries to the limbs [8,39]. In addition, sows are most commonly housed on fully slatted concrete floors (as was the case in the current study), which are rough and uncomfortable, and a risk factor for lameness [39,40]. The longer sows spend on this type of floor, the greater the likelihood of increased locomotion scores as a result of leg discomfort experienced by the animals. Provision of more comfortable floor surfaces, such as rubber mats, could help to reduce lameness throughout pregnancy [41]. Rubber mats/floors are associated with greater ease of changing posture [42], fewer foot and claw injuries, and are more comfortable to rest on [39]. A reduction in lameness can also be achieved through the provision of bedding such as straw, as bedding can minimise the negative impact of rough concrete floors on sow feet and claws [43,44]. Additionally, early detection of locomotion issues is crucially important when attempting to reduce lameness, as treatment applied early can be more effective [2,10].

This is the first study that we are aware of which investigated variation in individual components of gilt stride. Information on specific components presents a more detailed picture of locomotory ability as pregnancy progresses, and mirrors similar work with dairy cows [20,21]. These authors were able to attribute higher overall locomotion scores in a proportion of dairy cows to higher scores for individual locomotion components such as “tracking up” [21], and abduction/adduction [20]. More importantly, they were able to relate these differences back to specific hypotheses developed in relation to the experimental treatments; for instance, O’Driscoll et al. [20] hypothesised that more ab/adduction in cows milked once daily compared to twice daily was due to the legs swinging out around an engorged udder. Looking at specific components of locomotion could therefore provide insights into the underlying causes of lameness, help to ameliorate its risk factors, and could in turn be important when deciding on the best form of treatment.

Farmers are not trained to assess locomotion [37]. Locomotion scoring is complex, as to do so reliably usually requires observing several aspects of locomotion simultaneously, which is challenging even for trained personnel [37,45]. Thus, identification of a single locomotion component which could act as a reliable measure of the animal’s overall locomotory ability would therefore be extremely useful [45]. It could speed up and potentially make on-farm locomotion assessment more accurate by simplifying the methodology for the farmer [45]. As an example from the dairy cow literature, it is commonly accepted that the degree of back arch displayed by a cow provides insight into overall locomotory ability/lameness status, as the two are positively associated [45,46].

The current study identified caudal sway, stride length, fluidity of movement, and reluctance to bear weight while walking as being positively associated with the overall locomotion score assessed using a VAS, demonstrating potential to simplify sow locomotion assessment on-farm. However, when we compared patterns over time, and whether the relationship between OVERALL and each component on each day was similar, we found that caudal sway is likely not a suitable proxy for OVERALL locomotory ability. The lack of a positive association on each recording day could be a consequence of the changing weight and shape of the gilt as pregnancy progresses, which in turn could alter the degree of her caudal sway. A similar phenomenon was noted in dairy cows by O’Driscoll et al. [20], whereby the degree of abduction and adduction recorded in the animals differed depending on the fullness of their udders. A good proxy for OVERALL locomotion should have a consistent relationship with it across all stages of pregnancy and management. Stride length, fluidity of movement, and reluctance to bear weight while walking all had a consistent relationship with OVERALL across and on each scoring day separately, and thus have potential to be used as proxies for OVERALL locomotory ability.

While stride length requires a degree of familiarity and experience to be scored accurately [11,47], fluidity of movement and reluctance to bear weight while walking could be more appropriate options for farmers. Fluidity of movement is a measure of the overall smoothness/ease of an animal’s walking ability, where any deviations away from the norm are easy to observe. Reluctance to bear weight while walking requires the observer to identify whether the animal is reluctant to place any of its limbs on the floor, and the degree to which this occurs, to determine the severity of the phenomenon. Abnormal weight bearing is easily spotted; thus, similar to fluidity of movement, any deviations away from the norm are easy to identify. Further work consisting of repeatability testing involving producers, advisors, and vets should examine both of these aspects in more detail to determine the ease with which they can be learned, and thus ascertain their suitability for on-farm use.

Lameness has detrimental effects on reproductive performance, potentially mediated by chronic stress [1,4,48,49]. The current study found positive associations between the VAS locomotion score of gilts around their first service and the total number of piglets born, as well as a trend for a positive association with piglets born alive, over the first four parities. There was also a positive association between the VAS locomotion score at this time and the number of piglets born dead, and a trend for a positive association with mummified piglets. Locomotory ability later on was related to long-term reproductive performance to a much lesser extent, with just a trend for a positive association between the VAS locomotion score and the total born and born alive piglet numbers. Thus, it appears that assessing locomotory ability around the time of first service is likely the optimal time to estimate how it could affect lifetime performance.

As higher scores indicate worsening locomotory ability, this implies that gilts that deviated more from the ‘ideal’ stride around the time of first service were more productive across their first four parities. These findings conflict with the existing literature. In the studies of Anil et al. [8] and Iida et al. [50], lame sows had lower numbers of piglets born alive, thus demonstrating a detrimental effect of lameness on reproductive performance. However, these studies utilised locomotion scores recorded at different stages of pregnancy to those used in the current study (e.g., only on the way to the farrowing rooms). Moreover, it is possible that this difference to our findings relates to the fact that the above studies considered effects of clinical lameness, rather than a slight impairment in locomotion, as was the case in our study. It is possible that as clinical lameness is a more severe condition, this led to much higher chronic/acute stress levels, and consequently had a more marked effect on reproductive performance parameters [8,50].

A possible explanation for our findings regarding associations between locomotory ability and reproductive performance could relate to energy resource distribution in sows. Redirection of energy resources away from non-crucial physiological processes towards reproduction is a known phenomenon in mammals, as this strategy maximizes reproductive performance [51]. It is possible that our study sows redirected their energy resources towards reproductive functions in a likewise manner, with positive impacts on the number of total born and born alive piglets. In consequence, this could have left fewer energy resources available for the maintenance of leg health, resulting in slight deviations from optimal locomotion.

Following on from this, we speculate that the slightly compromised leg health (as marked by slight deviations from optimal locomotion) experienced even at this early stage in the reproductive cycle generated elevated stress levels, which persisted chronically. This is supported by our finding of higher hair cortisol concentrations in late pregnancy (reflecting chronic stress levels experienced by gilts throughout pregnancy) with higher overall locomotion scores both at service and in mid-pregnancy. The elevated stress levels could in turn have detrimental knock-on effects on prenatal mortality. Moreover, perhaps this could explain the positive association between locomotion scores at service and the numbers of piglets born dead, and the trend for a higher number of piglets mummified with increasing overall locomotion score. This finding is in line with Hartnett et al. [52]; in that study, replacement gilts reared alongside males had impaired leg health in terms of higher hoof lesion scores. These gilts went on to have higher numbers of piglets born dead over their first five parities, which the authors hypothesised was due to the elevated stress levels associated with impaired leg health [52]. Our finding is also in line with Pluym et al. [53], who found higher numbers of born dead and mummified piglets with an increasing incidence of claw lesions and wall cracks. Thus, it is possible that even slightly impaired leg health/locomotion could generate sufficient chronic stress levels to impair certain aspects of reproductive performance. Nevertheless, it is important to note that the fact that the study gilts had larger litter sizes in general could also explain the higher numbers of born dead and mummified piglets recorded in this study.

## 5. Conclusions

A detailed VAS developed in the current study was able to detect slight deviations from optimal gilt locomotion over time more effectively than a categorical scoring system. The extra information generated as a result of scoring of locomotion in terms of several locomotion components provided a greater insight into overall locomotory ability than was previously possible for sows. This should encourage the use of more detailed VAS scoring systems in the future, thus contributing to early detection and prevention of developing lameness disorders and simplifying on-farm locomotion assessment. Further work should apply the VAS developed in this study in locomotion scoring of older sows, and with multiple observers. Finally, this study pointed at the possibility of chronic stress resulting from impaired locomotion acting as a mediator for the process of reproductive performance impairment. Future research is necessary to further elucidate the mechanisms involved in the impairment of reproductive performance by slightly impaired locomotory ability, with a focus on the extent to which chronic stress associated with slightly impaired locomotion is involved in this process.

## Figures and Tables

**Table 1:** Details of experimental schedule and design.

Replicate	Replicate Size	Date of Mixing	Group Size at Mixing	Interval between Mixing Events (Weeks)
1	12	07/05/2019	33	9
2	10	28/05/2019	32	3
3	8	30/07/2019	19	9
4	4	20/08/2019	15	3
5	8	10/09/2019	21	3
6	4	22/10/2019	15	6
7	8	12/11/2019	17	3
8	4	03/12/2019	16	3

**Table 2:** Components of locomotion scored using a Visual Analogue Scale (VAS). The VAS ranged from 0 mm (perfect) to 150 mm (the most severe impairment possible).

Locomotion Component	Definition
Caudal sway	The side-to-side movement of the hindquarters
Stride length	The evenness of strides taken by the sow
Fluidity of movement	The overall ease with which the sow walks
Reluctance to bear weight while walking	Evidence of differences in weight bearing between the limbs, including shifting weight between hind/front legs, and intermittent placement of limbs on the floor
Abduction	Outward swinging of hind legs
Adduction	Inward swinging of hind legs

**Table 3:** Associations (regression coefficient and standard error; SE) between individual visual analogue scale (VAS) locomotion component scores and the VAS overall locomotion score in 51 gilts (n = 8 replicates), as a way of identifying a single locomotion component most associated with overall locomotory ability.

Individual Locomotion Component Score	Regression Coefficient	SE	F-Statistic	*p*-Value
Caudal sway	0.4	0.07	F_1,146_ = 32.23	<0.001
Stride length	0.7	0.02	F_1,147_ = 1090.77	<0.001
Fluidity of movement	0.8	0.04	F_1,147_ = 328.75	<0.001
Reluctance to bear weight while walking	0.8	0.04	F_1,147_ = 390.90	<0.001
Abduction	0.1	0.16	F_1,146_ = 0.20	0.652
Adduction	−0.02	0.649	F_1,146_ = 0.00	0.980

## Data Availability

The data presented in this study are available on request from the corresponding author.
